# Application of Pure and Ion-Doped FeB, CoB, MnB, and Fe2B Nanoparticles for Magnetic Hyperthermia

**DOI:** 10.3390/ma18122765

**Published:** 2025-06-12

**Authors:** Angel T. Apostolov, Iliana N. Apostolova, Julia M. Wesselinowa

**Affiliations:** 1 Department of Physics, University of Architecture, Civil Engineering and Geodesy, 1046 Sofia, Bulgaria; angelapos@abv.bg; 2 Faculty of Forest Industry, University of Forestry, 1756 Sofia, Bulgaria; inaapos@abv.bg; 3 Faculty of Physics, Sofia University, St. Kliment Ohridski, 1164 Sofia, Bulgaria

**Keywords:** magnetic hyperthermia, MnB and Co_2_B nanoparticles, ion doping, SAR coefficient

## Abstract

This study investigates Mn_1−x_X_x_B (X = Fe, Co) and (Fe_1−x_Co_x_)_2_B nanoparticles as candidates for self-controlled magnetic hyperthermia (SCMH) in cancer therapy. Using a microscopic model and Green’s function techniques, we calculate the Curie temperature, saturation magnetization, coercivity, and specific absorption rate as functions of nanoparticle size and dopant concentration. Surface and size effects are taken into account. The results are in good agreement with experimental data, confirming the model’s validity and highlighting the potential of these nanoparticles for efficient and safe magnetic hyperthermia applications. We have found that pure and doped MnB and Co_2_B nanoparticles with specific compositions meet biocompatibility requirements for SCMH suitable for in vivo and in vitro, for example, Mn_0.6_Co_0.4_B (*d* = 27.1 nm); Mn_0.5_Co_0.5_B (*d* = 32.2 nm); MnB (*d* = 26.3 m); (Fe_0.2_Co_0.8_)_2_B (*d* = 22.0 nm); (Fe_0.1_Co_0.9_)_2_B (*d* = 26.3 nm); and Co_2_B (*d* = 31.7 nm).

## 1. Introduction

Magnetic hyperthermia (MH) is a method used in the treatment of oncological diseases [1]. The localized heating of malignant tumors within the range of 41 °C to 46 °C leads to their complete disintegration while preserving healthy cells. This effect is due to the fact that tumor tissues are more sensitive to overheating compared to healthy tissues. MH is a technique for heating malignant formations through the use of magnetic materials. It is based on the simple principle that magnetic nanoparticles (MNPs), when interacting with an alternating electromagnetic field, “generate” heat [2]. MNPs can be directed to cancerous tissues using special binding and targeting agents (chemical functionalization), making the procedure selective and effective. Electromagnetic fields pass through the body without obstruction and generate heat only within tissues associated with MNPs, thereby enabling heat delivery at the cellular level [3,4]. A major challenge in the in vitro and in vivo applications of MH is the monitoring of the temperature field around the tumor, as exceeding the target temperature can lead to the irreversible denaturation of proteins in healthy tissues and the formation of necrosis, potentially resulting in fatal outcomes for patients [5,6].

A solution to this problem involves the use of MNPs (ferromagnetic and ferrimagnetic) with a magnetic phase transition temperature (Curie temperature—*T_C_*) within the range of 41 °C to 46 °C. Upon reaching *T_C_*, the nanoparticles undergo a transition from a magnetically ordered to a magnetically disordered (paramagnetic) state, causing the heating process to naturally cease. This approach is referred to as self-controlled magnetic hyperthermia (SCMH). This requirement is not related to the specific nature of tumors but rather to the necessity of not exceeding the heating temperature for healthy tissues. It is well known that, at temperatures above 42–46 °C, damage to healthy tissues occurs, leading to necrosis with lethal consequences for the patient [3,4].

In this context, the magnetic phase transition temperature becomes a necessary condition for the applicability of MNPs in the method of SCMH, as it ensures the protection of the patient’s life. Additional criteria for the suitability of MNPs for SCMH include the following: (a) A high saturation magnetization *M_S_* value, which enables a stronger response to the external magnetic field. (b) A high coercive field *H_C_* value, which enhances the efficiency of the thermal heating process. (c) A nanoparticle size smaller than 35 nm, allowing for transport through capillary blood vessels and thus enabling both in vivo and in vitro applications. (d) Non-toxicity and biocompatibility. (e) The frequency *f* and amplitude *h_max_* of the external alternating electromagnetic field must also be biocompatible. The magnetic field should be biologically safe and within human pain tolerance limits. This constraint is defined by the expression: $h_{max}*f<$ 6.2 × 10^7^ Oe/s (so called $h_{max}*f$ factor [7]).

To characterize the heating capability of tissues by MNPs, the so-called specific absorption rate (SAR) coefficient is introduced. The SAR measures the energy absorption per unit mass of an MNP probe [8]. It is defined as the absorbed thermal power normalized to the mass of the MNPs when subjected to an external alternating electromagnetic field with a given frequency *f* and amplitude *h_max_*. When MNPs are exposed to an alternating magnetic field, they release heat during a single cycle equal to the area enclosed by their hysteresis loop. Experimental studies have shown that the optimization of heating efficiency depends on several factors, including the particle size, saturation magnetization, coercivity, and the effective magnetic anisotropy constant. Moreover, the SAR exhibits a non-monotonic dependence on these parameters [9,10,11,12].

By modeling the heterogeneity of nanoparticles through modifications of the exchange interaction constants, magnetic anisotropy, and the number of nearest neighbors at the surface and in the core, we have conducted detailed investigations in our previous works on MNPs with structural formulas La_1−x_Sr_x_MnO_3_ (LSMO) [13] and Me_1−x_Zn_x_Fe_2_O_2_ (MZFO), where Me = Co, Ni, Cu, or Mn [14]. In this way, we determined a set of nanoparticles suitable for application in MH, based on their magnetic phase transition temperature, particle size, and magnetic characteristics.

Nanoparticles (NPs) of transition metal borides are promising magnetic materials for biomedical applications due to their characteristic chemical properties, biocompatibility, controllable size, and good dispersibility. By biocompatibility, we mean that these NPs must be non-toxic. On the other hand, these non-toxic MNPs must meet, from a physical point of view, several conditions, listed above (*T_C_*, size, and f × h_max_ < 6.2 × 10^7^ Oe/s), for applicability in magnetic hyperthermia, which must protect the human body from biodegradability. Iron boride (FeB) is one such compound, distinguished by its low toxicity, high stability, and tunable magnetic properties [15,16,17,18,19]. As reported by Aydemir et al. [15] and Hamayun et al. [19], a decrease in the nanoparticle size leads to a reduction in magnetization and an increase in coercive field. Furthermore, it has been established that FeB NPs exhibit a significant magnetothermal response under alternating magnetic fields, making them highly promising candidates for applications in MH [19].

Iron diboride (Fe_2_B) NPs are also considered as potential materials for biomedical applications and MH [20,21,22]. It should be emphasized that Fe_2_B exhibits higher spontaneous magnetization and a higher Curie temperature compared to FeB [21,22]. Studies by Mertdin-Uelkuseven et al. [20] on pure Fe_2_B NPs show a dependence of the SAR on particle size, with the heat transfer effect decreasing as the NP size decreases. First-principles studies on the Debye temperature, as well as the mechanical and magnetic properties of the Fe_2_B compound, have been presented by Wei et al. [23]. The authors perform a comparative analysis of the results for Fe_2_B with and without spin polarization.

Narita et al. [24] have shown that FeB nanoparticles exhibit higher resistance to oxidation and are stable when dispersed in aqueous solution. The low level of oxidation leads to an increase in the saturation magnetization [25], which allows better control of the nanoparticles in an externally applied magnetic field.

For biomedical applications, Fe_3_O_4_ or Fe_2_O_3_ are the most commonly used nanoparticles, but they have low chemical stability and are hard magnetic materials, whereas Fe–B compounds are typically soft magnetic materials with high chemical stability [17,26].

In [27], the authors confirm that Fe–B nanoparticles are biocompatible, which makes them suitable for in vivo and in vitro studies. It is claimed that iron boride nanoparticles are appropriate for biomedical applications due to their magnetic properties and their corrosion resistance associated with the boron content [16]. Fatima et al. [28] have studied the cytotoxicity of FeB nanoparticles encapsulated in liposomes. They show that these nanoparticles are biocompatible and are candidates for biomedical applications such as magnetic hyperthermia (MHT). Additionally, iron borides are economically advantageous.

Manganese boride (MnB) is a non-toxic and biocompatible material, and its NPs also show potential for use in MH [29]. The magnetic properties of MnB have been studied by Fu et al. [30], Adamski et al. [31], Jiang et al. [32], Kalyon et al. [33], and Ahmed et al. [34].

An important requirement for MNPs to be applicable in magnetic hyperthermia (MH) is their non-toxicity. Since this article does not investigate toxicity issues, we note that the biocompatibility of MNPs is enhanced by adding a special coating. Studies of MNPs “coated” with SiO_2_ [35], dextran [5], certain ligands [36], and fatty acids [37] demonstrated colloidal stability and biocompatibility.

Additionally, doped NPs of FeB, Fe_2_B, and MnB with various transition metal ions, such as Co, Ni, and Mn, are also being investigated for MH applications [38,39,40,41,42,43]. The introduction of these ions results in a reduction in magnetization and an increase in the coercive field. It has been established that MnB exhibits higher spontaneous magnetization and a higher coercive field compared to FeB [40]. Calculations of the magnetocrystalline anisotropy energy for the system (Fe_1−x_Co_x_)_2_B have been performed using density functional theory methods by Daene et al. [44].

In our previous articles, we have theoretically investigated the dependence of the SAR on the main static and dynamic properties of magnetic nanoparticles from a physical perspective [45,46]. Qualitative explanations for the observed SAR dependencies from a microscopic viewpoint were presented.

In this article, we apply these microscopic models and methods to specific pure and doped monoboride MNPs, obtaining quantitative values for magnetization, coercivity, size, and thermal efficiency (SAR coefficient).

## 2. Model and Method

**Figure 1:**
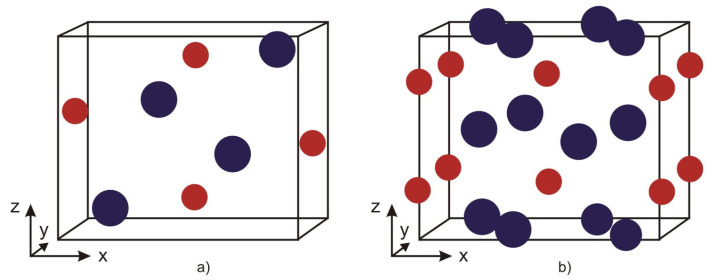
Crystal structures of (**a**) XB (X = Mn, Co, Fe) with space group Pnma and (**b**) X_2_B with space group I4/mcm. Blue circles denote X = Mn, Co, and Fe ions; red ones—B ions.

The compound XB, where X = Fe, Co, or Mn, crystallizes in an orthorhombic structure with space group *Pnma* (see Figure 1a). The boron atoms form zigzag chains along the *b*-axis, while isolated X atoms are located between them, arranged in triangular prisms [40]. These materials exhibit magnetic phase transition temperatures well above room temperature and possess outstanding ferromagnetic properties. These characteristics arise from the magnetic exchange interactions and correlations between the transition metal atoms, as well as from the covalent character and bond lengths of the transition metal–boron bonds. Upon the substitution of Mn ions in Mn_1−x_(Co, Fe)_x_B, the crystal structure of the lattice remains intact. In this process, the lattice parameters *a* and *c* decrease with increasing Co and Fe dopants, whereas the lattice constant along the *b*-axis increases with higher Co concentration and decreases with increasing Fe concentration. Overall, the changes along the *b*-axis are about an order of magnitude smaller than those observed along the *a*- and *c*-axes [39]. In MnB and FeB, the *c*(*z*)-axis corresponds to the easy axis of magnetization, indicating that, for XB compounds and their solid solutions, the first magnetic anisotropy constant K_1_ is positive [31].

The Hamiltonian describing the magnetic properties of Mn_1−x_X_x_B (X = Co, Fe) is

(1) $$H_{{Mn}_{1-x}X_{x}B}=-\left(1-x\right)\sum_{ij}J_{ij}^{Mn-Mn}{\vec{S}}_{i}^{Mn}.{\vec{S}}_{j}^{Mn}-y\sum_{ik}J_{ik}^{Mn-X}{\vec{S}}_{i}^{Mn}.{\vec{S}}_{k}^{X}-\;x\sum_{kl}J_{kl}^{X-X}{\vec{S}}_{k}^{X}.{\vec{S}}_{l}^{X}--\left(1-x\right)\sum_{i}K_{i}^{Mn}{\left(S_{i}^{z,Mn}\right)}^{2}-x\sum_{i}K_{i}^{X}{\left(S_{i}^{z,X}\right)}^{2}-(1-\;x)\sum_{i}\vec{h}.{\vec{S}}_{i}^{Mn}-x\sum_{i}\vec{h}.{\vec{S}}_{i}^{X},$$

where y = x for x ꞓ [0–0.5], and y = 1 − x for x ꞓ [0.5–1.0].

${\vec{S}}_{i}^{Mn}\;и\;{\vec{S}}_{j}^{X}$, (X = Co; Fe), are the Heisenberg spin operators for the Mn ions and the dopant ions, respectively. $J_{ij}^{Mn-Mn}>0\;;\;J_{ik}^{Mn-X}>0\;и\;J_{kl}^{X-X}$ > 0 are the exchange interaction constants. The first term describes the ferromagnetic coupling between the spins of the Mn ions, while the second and third terms account for the coupling between ${Mn}^{3+}-X^{3+}$ and $X^{3+}-X^{3+}$ magnetic ions, respectively. ${K_{i}}^{Mn,X}>0$ denotes the single-ion anisotropy constants. $\vec{h}$ is an applied magnetic field. Let us emphasize that the exchange interaction constant J_ij_ = J(r_i_ − r_j_) depends inversely proportional to the square of the distance between two neighboring spins, ($J\sim \frac{1}{r^{2}}$), i.e., to the lattice parameters. This distance changes upon doping due to the difference in ionic radii between the host and dopant ions, which significantly influences the value of the exchange interaction.

X_2_B (X = Fe, Co), as well as the doped compounds (Fe_1−x_Co_x_)_2_B, crystallize in a body-centered tetragonal structure with space group I4/mcm, characterized by alternating layers of iron and boron ions (see Figure 1b). Two of the Fe–B bonds are shorter, while the other two are longer compared to those in a body-centered cubic unit cell. The unit cell contains four equivalent X atoms at positions with point symmetry group mm, and two equivalent B atoms at positions with point symmetry group 42 [47]. In X_2_B, the B atoms are located between two layers of Fe atoms arranged in a distorted close-packed configuration, as shown in Figure 1b. As the concentration of dopant Co ions increases, the volume of the crystal lattice decreases.

Magnetic X_2_B compounds exhibit nearly isotropic behavior between 70 K and 593 K, with the magnetization vector lying within the (001) easy magnetization plane. Below 70 K in Co_2_B, and above 593 K in Fe_2_B, the materials transition to uniaxial magnetic anisotropy, where the *c*-axis becomes the easy axis of magnetization [41]. Experimental investigations [48] reveal that, near the room temperature, in doped (Fe_1−x_Co_x_)_2_B samples, the magnetic anisotropy constant *K* is positive for compositions x ∈ [0.1, 0.5], indicating an easy axis along *z*(*c*). Conversely, for x ∈ [0.6, 1.0], *K* becomes negative, implying that the easy magnetization plane is oriented perpendicular to the *z*(*c*)-axis. Thus, a strong dependence of both the magnitude and the sign of the magnetocrystalline anisotropy on Co doping concentration is observed. For the purposes of the present study, spin reorientation processes can be neglected [41].

The Hamiltonian describing the magnetic properties of (Fe_1−x_Co_x_)_2_B is given by

(2) $$H_{{\left({Fe}_{1-x}{Co}_{x}\right)}_{2}B}=-2\left(1-x\right)\sum_{ij}J_{ij}^{Fe-Fe}{\vec{S}}_{i}^{Fe}.{\vec{S}}_{j}^{Fe}-2y\sum_{ik}J_{ik}^{Fe-Co}{\vec{S}}_{i}^{Mn}.{\vec{S}}_{k}^{Co}-\;2x\sum_{kl}J_{kl}^{Co-Co}{\vec{S}}_{k}^{X}.{\vec{S}}_{l}^{X}--2\left(1-x\right)\sum_{i}K_{i}^{Fe}{\left(S_{i}^{z,Fe}\right)}^{2}-2x\sum_{i}K_{i}^{Co}{\left(S_{i}^{z,Co}\right)}^{2}-2(1-\;x)\sum_{i}\vec{h}.{\vec{S}}_{i}^{Fe}-2x\sum_{i}\vec{h}.{\vec{S}}_{i}^{Co},$$

where y = x for x ꞓ [0–0.5], and y = 1 − x for x ꞓ [0.5–1.0].

${\vec{S}}_{i}^{Fe}\;and\;{\vec{S}}_{j}^{Co}$ are the Heisenberg spin operators for Mn and Co ions, respectively. $J_{ij}^{Fe-Fe}>0\;;\;J_{ik}^{Fe-Co}>0\;and\;J_{kl}^{Co-Co}$ > 0 are the exchange interaction constants. ${K_{i}}^{Fe,Co}<0$ is the single-ion anisotropy constant, indicating that the (001) plane is the easy magnetization plane. $\vec{h}$ represents a constant external magnetic field.

To achieve the objectives of the present study, we need to calculate the magnetization, coercivity, and the SAR coefficient, which quantifies the conversion of magnetic energy into heat during the process of remagnetization of MNPs under an external alternating electromagnetic field with a given frequency *f* and maximum amplitude *h_max_*. As previously mentioned, MNPs suitable for hyperthermia applications must meet specific requirements for the values of the Curie temperature *T*_C_, coercive field *H_c_*, saturation magnetization *M_s_*, and particle size *d*. These magnetic and dimensional properties determine the thermal parameters of SAR for the NPs. From a microscopic perspective, these magnetic characteristics are size-dependent; i.e., they are influenced by the shell/core ratio and by size and surface effects. These effects lead to modifications in the magnetic interactions and surface anisotropy compared to the bulk material, as well as changes in the number of nearest neighbors due to the loss of translational symmetry and surface oxidation processes. Consequently, the magnetic properties of NPs can be manipulated through size variation, doping, and specific surface treatments.

To determine the characteristics outlined above, we will perform calculations at the microscopic level, incorporating both the static and dynamic properties of the system. In this study, we will calculate the SAR based on a microscopic model, analyzing how its values depend on the microscopic parameters of the system and the composition of the NPs, while accounting for their heterogeneity and ensuring compliance with biocompatibility requirements.

For this purpose, we will employ the *Kubo formalism*, which describes the system’s response to a time-dependent perturbation. For clarity, we will consider a linearly polarized alternating magnetic field acting on the NPs. The average absorbed power (the energy absorbed per unit time) *P* for the MNP is given by the following:

(3) $$P=-2\sum_{\begin{array}{c} p,q\\ \omega >0 \end{array}}\omega \chi_{pq}^{\prime \prime }\left(\omega \right)h_{max}^{p}h_{max}^{q},\;p,q=x,\;y,\;z,$$

where *χ*_*pq*_″(*ω*) is the imaginary part of the magnetic susceptibility, and, at the microscopic level, it is expressed through the retarded Green’s function. For an MNP, the magnetic susceptibility takes the form:

(4) $$\chi_{pq}\left(\omega \right)=-\sum_{\begin{array}{c} i,i\\ \alpha ,\beta \end{array}}g^{2}\mu_{B}^{2}{\ll {\left({\vec{S}}_{i}^{\alpha }\right)}^{p};\;{\left({\vec{S}}_{j}^{\beta }\right)}^{q}\gg }_{\omega },$$

where *α*, *β* are the spin variables in the defined sublattices, and *i*, *j* denote the summation over the nearest neighbors. *ω* = 2*πf*, *g* is the gyromagnetic ratio, and *μ*_*B*_ is the Bohr magneton. From Equation (3), the area of the hysteresis loop can be computed using quantities that characterize the system from a microscopic perspective. This enables us, based on the defined model Hamiltonian, to calculate the required retarded Green’s functions ${\ll {\left({\vec{S}}_{i}^{\alpha }\right)}^{p};\;{\left({\vec{S}}_{j}^{\beta }\right)}^{q}\gg }_{\omega }$ using the Tserkovnikov method [49]. From the expression *SAR* = *Pfρ*, the dependence of the thermal heating efficiency on the microscopic characteristics of the magnetic system can be determined.

If the linearly polarized magnetic field is applied along the *x*-axis, to calculate the absorbed power *P*, we need to compute the transverse susceptibility *χ*^*xx*^(*χ*_⊥_). The relationship between the transverse susceptibility and the absorbed power is crucial for understanding the energy dissipation process in the system.

(5) $$\chi^{xx}=\chi_{⊥}=-\sum_{\begin{array}{c} i,i\\ \alpha ,\beta \end{array}}g^{2}\mu_{B}^{2}{\ll {\left[{{\vec{S}}_{i}}^{\left(X^{\alpha }\right)}\right]}^{x};\;{\left[{{\vec{S}}_{j}}^{\left(X^{\beta }\right)}\right]}^{x}\gg }_{\omega },$$

If, instead of ${\left({\vec{S}}_{i}^{X}\right)}^{x};\;{\left({\vec{S}}_{i}^{X}\right)}^{y};\;{\left({\vec{S}}_{i}^{X}\right)}^{z}$, we introduce the operators ${\left(S_{i}^{X}\right)}^{\pm }$ = ${\left({\vec{S}}_{i}^{X}\right)}^{x}\pm i{\left({\vec{S}}_{i}^{X}\right)}^{y}$, and considering that ⟪+;−⟫_*ω*_ = ⟪−;+⟫_−*ω*_, we introduce the following retarded Green’s functions for calculating the transverse susceptibility:

(6) $$G_{ij,E}^{Mn-Mn}{=\ll {\left(S_{i}^{Mn}\right)}^{+};\;{\left(S_{j}^{Mn}\right)}^{-}\gg }_{E}\;G_{ij,E}^{X-X}{=\ll {\left(S_{i}^{X}\right)}^{+};\;{\left(S_{j}^{X}\right)}^{-}\gg }_{E}\;\;\;G_{ij,E}^{Mn-X}{=\ll {\left(S_{i}^{Mn}\right)}^{+};\;{\left(S_{j}^{X}\right)}^{-}\gg }_{E}\;\;G_{ij,E}^{X-Mn}{=\ll {\left(S_{i}^{X}\right)}^{+};\;{\left(S_{j}^{Mn}\right)}^{-}\gg }_{E}\;$$

with X = Co, Fe.

To incorporate damping effects for definiteness, we will assume that Green’s functions have poles in the lower part of the complex plane, i.e, $E_{ij}^{\sim }$ = ±*E*_*ij*_ − *iγ*_*ij*_, where *γ*_*i*_ is the damping. Green’s functions, the energy of the elementary excitations, and the damping are calculated using the Tserkovnikov method [36] and given in Appendix A.

The magnetization for Mn_1−x_X_x_B (X = Co, Fe) is calculated using the formula:

(7) $$M_{S}=\left(1-x\right)M^{Mn}+xM^{X},$$

where $M^{X}=\frac{1}{N^{X}}\sum_{i}<{\left(S_{i}^{X}\right)}^{z}>$, as $<{\left(S_{i}^{X}\right)}^{z}>$ is determined from the following expression:

(8) $$<{\left(S_{i}^{X}\right)}^{z}>=\left[S^{X}+0.5\right]coth\left\{\left[S^{X}+0.5\right]\beta \omega_{i}(S^{X})\right\}-0.5coth\left[0.5\beta \omega_{i}(S^{X})\right],$$

with *β* = 1/*k*_*B*_*T* and $\omega_{i}(S^{X}$) = $\frac{1}{N^{X}}\sum_{j}\omega_{ij}(S^{X})$.

$\omega_{ij}(S^{X}$) are calculated beyond the random phase approximation (RPA), i.e., taking into account the following correlation functions: $<{\left(S_{i}^{Mn}\right)}^{-};\;{\left(S_{j}^{Mn}\right)}^{+}>$; $<{\left(S_{i}^{X}\right)}^{-};\;{\left(S_{j}^{X}\right)}^{+}>$; $<{\left(S_{i}^{Mn}\right)}^{-};\;{\left(S_{j}^{X}\right)}^{+}>$ and ${<\left(S_{i}^{X}\right)}^{-};\;{\left(S_{j}^{Mn}\right)}^{+}>$. They are obtained using the Spectral Theorem [50] and are given in Appendix A. In this way, we ensure self-consistency when calculating the static and dynamic properties of the considered system. The expressions for $\omega_{ij}(S^{X}$) and the average absorbed power P are given in Appendix B and Appendix C, respectively.

Even compositionally homogeneous NPs are structurally heterogeneous, because the surface atoms respond differently to external magnetic fields compared to those in the core. From a microscopic perspective, this leads to a modification in the character of spin interactions at the surface, within the core, and between them, significantly affecting the magnetic properties of the NPs. As the NP size decreases, the influence of the surface shell becomes increasingly pronounced due to the changed surface-to-volume ratio.

Therefore, an NP is conceptually divided into two regions: a core and a shell. The core consists of magnetic ions whose nearest neighbors are similar to those found in bulk materials. The shell surface consists of spins with a reduced number of nearest neighbors due to the broken translational invariance at the NP’s “edge.”

For quantitative calculations, we define the exchange interactions within the core as *J_b_* and at the surface of the MNP as *J_s_* with *J_s_* = 0.85 *J_b_*. The choice of a smaller exchange interaction constant at the shell compared to the core of the NPs is based on the fact that the saturation magnetization of NPs is lower than that of bulk samples [18]. Within the framework of the Heisenberg Hamiltonian, we also assume the presence of single-ion magnetic anisotropy, with different values in the core *K_b_* and at the surface *K_s_* as *K_s_* = 0.1 *K_b_*, according to experimental data for FeB [19]. This assumption reflects the negligible contribution of surface anisotropy in these NPs.

In this model, the magnetization is calculated taking into account the crystal structure, where deviations are considered through the reduction in the number of nearest neighbors and modifications in the exchange constants. The exchange constants (*J_s_*, *J_b_*) can be conveniently adjusted to accurately describe the magnetization of the NPs and the temperature of the magnetic phase transition. Similarly, introducing modifications to the interactions upon doping allows for the description of the dependence of both static and dynamic properties on the degree of doping.

Within the framework of our model, we assume that the MNPs are spherical, possess identical sizes (i.e., the probe is monodisperse), and do not interact with each other.

When considering doping, it is assumed that secondary phases and clusters of dopant ions do not form.

In [16] it is shown that the magnetic interactions between FeB nanoparticles are very weak. It has been established that the ratio between the remanent magnetization and the saturation magnetization is less than 0.5. This indicates that the MNPs are not bulk-coupled; i.e., the magnetic interactions between FeB nanoparticles are very weak, and they do not form ferromagnetic clusters. This means that aggregation between them in the sample could be neglected.

It should be noted that, since the polydispersity, i.e., the size variation in the MNPs within a given probe, is mainly influenced by the synthesis method [51,52], this issue has not been addressed. Within the current model, dipole–dipole interactions are not discussed or considered.

For the calculation of the SAR coefficient, we will work with the so-called standard probe, characterized by an NP concentration of 10 mg/mL, which, according to McBride et al. [53], represents the optimal concentration for magnetic hyperthermia therapy.

## 3. Numerical Calculation and Discussion

### 3.1. Bulk Mn_1−x_X_x_B (X = Co, Fe)

Let us note that we begin with the presentation of the magnetic characteristics of the bulk samples and the temperature of their magnetic phase transition as a function of the concentration of the dopant elements aims to demonstrate and confirm the adequacy of our microscopic model. The availability of sufficient experimental data for the bulk materials allows us to determine whether there is quantitative agreement between experiment and theory and, if necessary, to make corrections to the model and its parameters by the later consideration of nanoparticles.

For MNPs, due to the lack of repeatable experimental results, the absence of such results, or the impossibility of comparison, such verification is difficult to establish. This peculiarity of experimental results with nanoparticles is determined by the fact that their magnetic characteristics depend on many factors: the method of nanoparticle growth, size, shape or deviations from shape, different values and frequencies of external magnetic fields, the material used for “coating” the particle, and others.

For the numerical calculations of bulk Mn_1−x_X_x_B (X = Co, Fe), we use the following model parameters:

The exchange interaction constants in the undoped compounds are calculated within the framework of the mean-field approximation, using the following expression: ${\;J}^{X-X}=\frac{3\;{T_{C}}^{X}}{2zS\left(S+1\right)}$ in units of K, where ${Tc}^{X}$ is the magnetic phase transition temperature listed in column 5 of Table 1, and *z* is the number of nearest neighbors. In the doped samples, the exchange interactions between Mn ions and the dopant ions are *J*^Mn-Co^ = 24.51 K и *J*^Mn-Fe^ = 53.34 K. In the study of [25], using spin-polarized density functional theory (DFT) within the generalized gradient approximation (GGA), the following estimate for $J^{Mn-Mn}=35.96\;K$ was obtained, which is close in value to our calculated result.

We calculate J^Mn-X^ (X = CO; Fe) in units of Kelvin from the following equation:

$$J^{Mn-X}=\frac{3\;}{2z}\sqrt{\frac{\left(Tc-\left(1-x\right)T_{C,Mn})(Tc-\left(x\right)T_{C,X}\right)}{x(1-x)S_{Mn}(S_{Mn}+1)S_{X}(S_{X}+1)}}$$

where $T_{C,Mn}$ is the magnetic phase transition temperature for MnB; $T_{C,X}$ is the magnetic phase transition temperature for XB, with X = Co; and the values of Fe are given in Table 1 and Table 3. Tc is the Curie temperature for a given concentration x of the dopant ions in Mn_1−x_Fe_x_B and Mn_1−x_Co_x_B, which can be taken from references [54,56], respectively.

First, we will present the dependence of the magnetic phase transition temperature *T_c_*, the saturation magnetization *M_S_*, and the coercivity *H_C_* as a function of the doping level *x* for bulk Mn_1−x_Co_x_B and bulk Mn_1−x_Fe_x_B.

**Figure 2:**
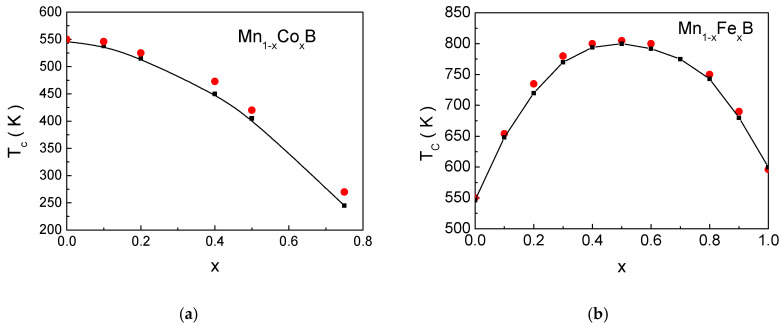
Dependence of *T_C_* on the concentration *x* of the dopant magnetic ions for (**a**) bulk Mn_1−x_Co_x_B and (**b**) bulk Mn_1−x_Fe_x_B. The red points correspond to experimental values from [54] for Mn_1−x_Fe_x_B and from [56] for Mn_1−x_Co_x_B.

Figure 2 shows the dependence of the phase transition temperature *T_C_* on the concentration *x* for bulk materials with (a) Co and (b) Fe ion doping. Qualitatively, the decrease in *T_C_* with Co is explained as follows: As is well known, the value of the magnetic phase transition temperature depends on the strength of the exchange interaction between magnetic spins. The exchange interaction J_Mn−Mn_ has the highest intensity compared to J_Mn−Co_ = 0.84 J_Mn−Mn_ and J_Co−Co_ = 0.7 J_Mn−Mn_. With the increase in Co ions, the number of pairings between Mn-Co and Co-Co ions increases at the expense of Mn-Mn pairings, which leads to a decrease in the effective exchange interaction between magnetic ions and thus to a lowering of the phase transition temperature. The decrease in the phase transition temperature with increasing Co dopant concentration has also been observed in other doped compounds [57,58].

The case of MnB doped with Fe ions is more interesting. Here, *T_C_* increases with the addition of Fe atoms for 0.0 < *x* < 0.5, reaching a maximum, after which, with further increase in *x*, the phase transition temperature decreases.

The results obtained can be qualitatively explained as follows: As the concentration of Fe ions increases, the number of Mn–Fe interactions, which are the strongest among the exchange interactions, also increases. This number reaches a maximum at *x* = 0.5, resulting in a corresponding increase in *T_C_*. The subsequent decrease in *T_C_* with further doping is attributed to the reduction in the number of Mn–Fe interactions. The red circles in the figures represent experimental values of *T_C_* from references [54,56]. The good agreement between the calculated and experimental data provides strong evidence for the validity of the microscopic model proposed in this work.

**Figure 3:**
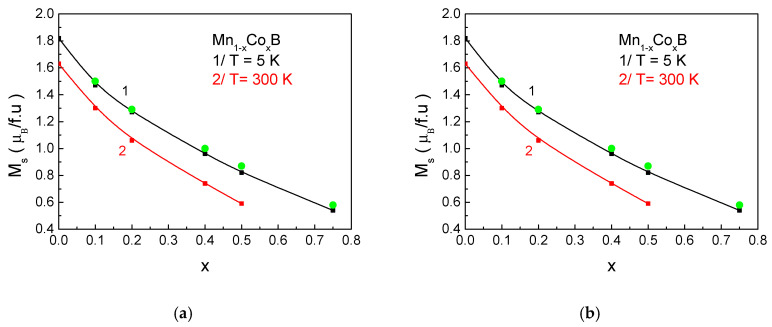
Dependence of *M_S_* on the impurity concentration *x* for (**a**) bulk Mn_1−x_Co_x_B and (**b**) bulk Mn_1−x_Fe_x_B at 1/*T* = 5 K and 2/*T* = 300 K. The green points represent experimental data for *M_S_* from [31,54].

Figure 3 presents the dependence of the magnetization *M_S_* on the impurity ion concentration *x* for (a) Mn_1−x_Co_x_B and (b) Mn_1−x_Fe_x_B at two temperatures: 5 K and 300 K. At low temperatures (*T* = 5 K), the increase in Co and Fe ion concentration leads to a decrease in the magnetization value. This reduction can be explained by the smaller magnetic moments of the Co and Fe impurities. In Mn_1−x_Co_x_B, Co atoms possess a significantly smaller magnetic moment compared to Mn atoms, which accounts for the sharp decrease in magnetization with increasing *x*. The Mn–Mn atomic distances are strongly influenced by the reduction in the lattice constant along the *c*-axis. The decrease in the distance between Mn ions leads to a broadening of the electronic bands and, consequently, to a reduction in the net magnetic moment. Similar effects have been theoretically predicted for MNPs [16].

At room temperature, a peak in the magnetization is observed in Mn_1−x_Fe_x_B at a dopant concentration of *x* = 0.3 (see Figure 3b, curve 2). This can be explained by the fact that, as the concentration of Fe ions increases, the internal molecular field (the effective exchange interaction in the compound) also increases. This leads to the suppression of thermal fluctuations, resulting in an initial increase in magnetization, since these fluctuations cause less deviation of the spins from the easy magnetization axis with increasing *x* at room temperature. However, with a further increase in *x*, the weaker exchange interaction between Fe–Fe at a fixed temperature leads to a decrease in the net magnetic moment. This type of behavior of magnetization as a function of impurity concentration is also observed in La_1−x_Sr_x_MnO_3_ [13]. Numerical calculations show good agreement with experimental results [31,55].

**Figure 4:**
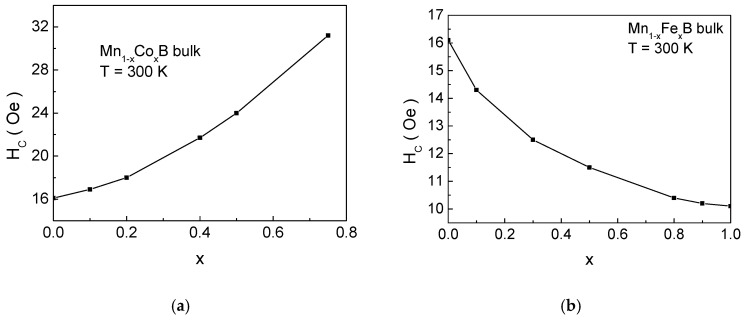
Dependence of *H_C_* on the impurity concentration *x* for (**a**) bulk Mn_1−x_Co_x_B and (**b**) bulk Mn_1−x_Fe_x_B at *T* = 300 K.

Figure 4 presents the dependence of coercivity *H_C_* on the impurity ion concentration *x* for (a) Mn_1−x_Co_x_B and (b) Mn_1−x_Fe_x_B. For Co doping, the coercivity increases, while, for Fe doping, it decreases. This is due to the fact that the anisotropy of Co ions is greater than that of Mn, while the anisotropy of Fe cations is lower than that of Mn ions. It is important to note that the total anisotropy of the formula unit is the relative sum of the anisotropies of Mn and the impurity ions. Consequently, when Mn is substituted by Co, the magnetocrystalline anisotropy increases, leading to an increase in *H_C_*, whereas substituting Mn with Fe results in a decrease in coercivity.

### 3.2. Mn_1−x_X_x_B (X= Co, Fe) Nanoparticles

As emphasized in the Introduction, the heating of tissues should not exceed temperatures above 46 °C. Such temperatures would lead to the irreversible denaturation of proteins in healthy cells, the formation of necrosis, and potentially a fatal outcome for the patient. Therefore, temperature control during heating is essential. For MNPs in a magnetically ordered state, this can be achieved if the transition temperature from a magnetically ordered to a magnetically disordered state is within the range of 312–315 K. Transitioning the particle to a paramagnetic state ensures the absence of hysteresis losses, which are responsible for transferring energy from the alternating electromagnetic field into heat. In this case, the process is self-regulating, preventing overheating. This is why the requirement for MNPs to have a magnetic phase transition temperature within the biocompatible range of 312–315 K is a necessary condition for their applicability in MHT for cancer treatment. It should also be noted that the transport of these particles to the sites of cancer cells or tumors is carried out through the circulatory system, which imposes another requirement on the size of the NPs. They should be no larger than 35 nm, ensuring their suitability for both in vitro and in vivo applications.

**Figure 5:**
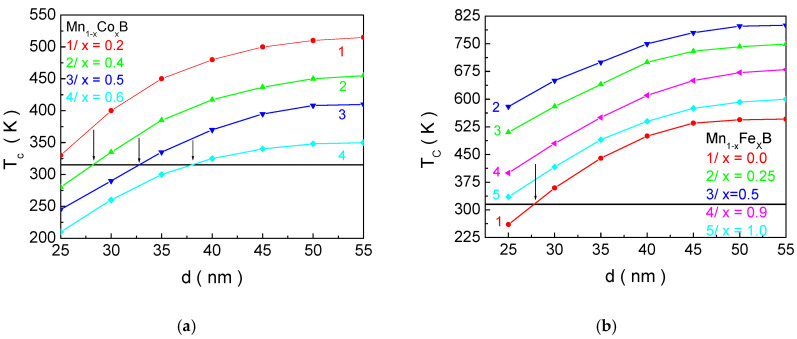
Dependence of the magnetic phase transition temperature *T_C_* on the NP size *d* for (**a**) Mn_1−x_Co_x_B and (**b**) Mn_1−x_Fe_x_B for different doping ion concentrations *x*.

Therefore, Figure 5 shows the dependence of the magnetic phase transition temperature *T_C_* for Mn_1−x_Co_x_B (Figure 5a) and Mn_1−x_Fe_x_B (Figure 5b) as a function of the NP size *d* for different concentrations of doping ions. The horizontal black line represents the boundary for *T_C_* (315 K), which should not be exceeded. As the NP size decreases, *T_C_* also decreases, regardless of the chemical composition and the concentration of doping elements. This can be explained by the change in the surface-to-volume ratio. As *d* is reduced, this ratio increases, meaning that the surface will have a greater influence on the magnetic properties of the NPs.

Since the exchange interaction in the shell is weaker than in the bulk (according to our model), this leads to a decrease in *T_C_*. The crossing of the *T_C_*(*d*) curves at different concentrations *x* of the doping element allows for the determination of the structural formula and size that satisfy the necessary condition for *T_C_* and the size for in vitro and in vivo applications of MNPs for MH. Based on this, we determine that NPs Mn_0.6_Co_0.4_B with *d* = 27.1 nm; Mn_0.5_Co_0.5_B—*d* = 32.2; and MnB—*d* = 26.3 nm are suitable candidates for MHT. For MNPs with the composition Mn_0.4_Co_0.6_B, the crossing of the biocompatibility boundary with the *T_C_*(*d*) curve (Figure 5a, curve 4) is observed, but in this case, the size of the NPs is 37.6 nm, which exceeds the requirement for in vitro and in vivo applications. According to our model, the size below which particles transition to a superparamagnetic state is 18.9 nm. Curve 1 in Figure 5a and curve 5 in Figure 5b satisfy the condition that *T_C_* should be below 315 K for sizes *d* = 20.9 nm and *d* = 19.8 nm, respectively. These sizes are close to the threshold for the onset of superparamagnetism established in the model (below this threshold, magnetic hysteresis is absent). Although they are slightly above this size, they are very close to it, and as the particles grow, their size distribution will result in insufficient sample efficiency (some particles will not transfer heat effectively). This would require the patient to undergo a longer exposure to a high-frequency electromagnetic field, which is unjustified, even though the *f. h_max_* factor is biocompatible. This is the reason why Mn_0.8_Co_0.2_B and FeB are not considered suitable for MH.

**Figure 6:**
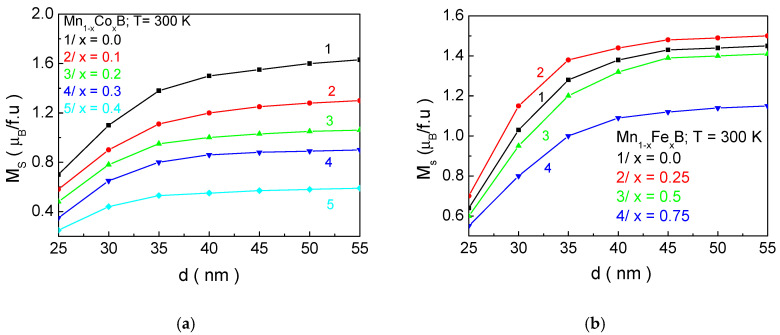
Dependence of the saturation magnetization *Ms* on the nanoparticle size *d* at *T* = 300 for (**a**) Mn_1−x_Co_x_B and (**b**) Mn_1−x_Fe_x_B at different concentrations *x* of the doping ions.

Figure 6 shows the dependence of the saturation magnetization *Ms* as a function of the nanoparticle size *d* for (a) Mn_1−x_Co_x_B and (b) Mn_1−x_Fe_x_B at different concentrations of the doping ion *x*. This behavior can be qualitatively explained as follows: At the surface, the translational invariance is disrupted, leading to a disruption in the number of nearest neighbors. Unpaired electron orbitals appear, and anharmonic contractions increase. Oxidation processes and implantation defects are observed. This leads to an increase in the lattice constants at the surface compared to the bulk. On the other hand, the exchange interaction constants depend on the distance between the spins, i.e., on the lattice parameter, the symmetry of the lattice, and the number of nearest neighbors. This will result in a decrease in the exchange interaction in the surface layer compared to the core for the considered compounds. The reduction in the exchange interaction constant at the surface *J_S_* leads to a lower magnetization of the surface layer compared to the core, and, at temperatures close to the magnetic phase transition temperature, the surface layer becomes practically non-magnetic. Therefore, with a decrease in the particle size, the influence of the surface layer increases, leading to a decrease in the magnetization of the magnetic nanoparticles.

For the NPs with the parameters Mn_0.6_Co_0.4_B—*d* = 27.1 nm; Mn_0.5_Co_0.5_B—*d* = 32.2 nm; and MnB—*d* = 26.3 nm, which are suitable candidates for hyperthermia, numerical calculations determine the following values for the saturation magnetization *M_s_*: Mn_0.6_Co_0.4_B—0.32 μ_B_/f.u; Mn_0.5_Co_0.5_B—0.23 μ_B_/f.u; and MnB—0.95 μ_B_/f.u.

**Figure 7:**
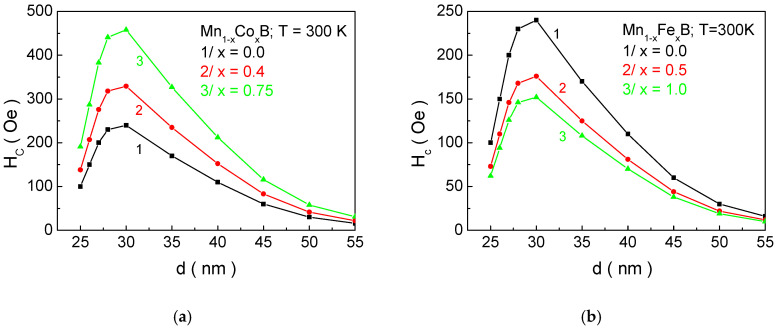
Dependence of the coercivity *H_c_* on the NP size *d* at *T* = 300 K for (**a**) Mn_1−x_Co_x_B and (**b**) Mn_1−x_Fe_x_B at different concentrations *x* of the doping ions.

Figure 7 shows the dependence of coercivity *H_c_* as a function of the nanoparticle size *d* for a/Mn_1−x_Co_x_B and b/Mn_1−x_Fe_x_B at different concentrations of the doping ion *x*. As the size of the NPs decreases, *H_c_* increases, reaching a maximum at d ~ 30 nm, after which it decreases. For sizes below 18.9 nm, coercivity becomes zero. The appearance of a peak in the dependence of *H_c_* on *d* is related to the transition from multi-domain to single-domain nanoparticles. For FeB, it has been established in the study of [9] that single-domain NPs have sizes below 30 nm, which shows good agreement with our theoretical results. Qualitatively, the decrease in coercivity with decreasing NP size in the single-domain region can be explained as follows: In the case of single-domain NPs, the energy barrier separating two energetically equivalent magnetic orientations is small. This means that it is possible to change the magnetic orientation even at low temperatures due to the low activation energy. Therefore, coercivity decreases with decreasing particle size in single-domain NPs. As the size decreases, thermal fluctuations increasingly compete with the energy of magnetic anisotropy and exchange energy. Below a certain size, thermal fluctuations dominate over magnetic anisotropy energy. The particles transition into a superparamagnetic state, and a magnetic hysteresis curve is not observed; i.e., coercivity becomes zero. From Figure 6, it can be seen that, as the concentration of Co ions in MnB increases, *H_c_* increases, while doping with Fe atoms leads to a decrease. The reason for this is that the anisotropy of Co ions is higher compared to that of Mn, while the anisotropy of Fe ions is lower. Within our model (as outlined in Section 2), the total anisotropy is equal to the relative sum of the anisotropies of Mn and X ions. Therefore, when substituting manganese ions with Co (Fe) ions, the magnetocrystalline anisotropy will increase (decrease) with increasing doping concentration, which will lead to an increase (decrease) in *H_c_* with increasing *x*. For NPs with parameters Mn_0.6_Co_0.4_B—*d* = 27.1; Mn_0.5_Co_0.5_B—*d* = 32.2 nm; and MnB—*d* = 26.3 nm, which are suitable candidates for hyperthermia, numerical calculations determine the following values of coercivity *H_c_*: Mn_0.6_Co_0.4_B—278 Oe; Mn_0.5_Co_0.5_B—352 Oe; and MnB—210 Oe.

The SAR coefficient depends on external parameters (the amplitude of the alternating magnetic field $h_{max}$ and its frequency *f*) and internal parameters: (1) the structure of the NPs (size, crystal structure, and doping); (2) magnetic properties (magnetic anisotropy, magnetization, coercivity). The efficiency of heat transfer from the NPs to the tissue also depends on the method of NP growth and annealing time, the polydispersity of the samples, and the dipole interaction between particles at high NP concentrations in the sample.

We will present an investigation of the SAR for NPs that meet the condition for biological compatibility of the magnetic phase transition temperature, ensuring the avoidance of tissue overheating. We will evaluate the following: (1) The thermal efficiency of NPs for undoped MnB and doped MnB with Co and Fe ions. The compounds that meet the parameters *T_c_*, *M_s_*, and *H_c_* will be considered suitable for their use in MH. (2) Whether the particles, at which the SAR reaches a maximum, are suitable for cancer treatment through magnetic heating. Therefore, we will study the dependence of the SAR on the size *d* for the following NPs: Mn_0.6_Co_0.4_B—*d* = 27.1 nm; Mn_0.5_Co_0.5_B—*d* = 32.2 nm; and MnB—*d* = 26.3 nm. Again, we emphasize that particles with sizes close to the superparamagnetic state will be considered unsuitable because, during their synthesis, there is a size distribution, and the efficiency of the probe decreases. The SAR calculation will be based on the microscopic model proposed by Apostolova et al. [45].

**Figure 8:**
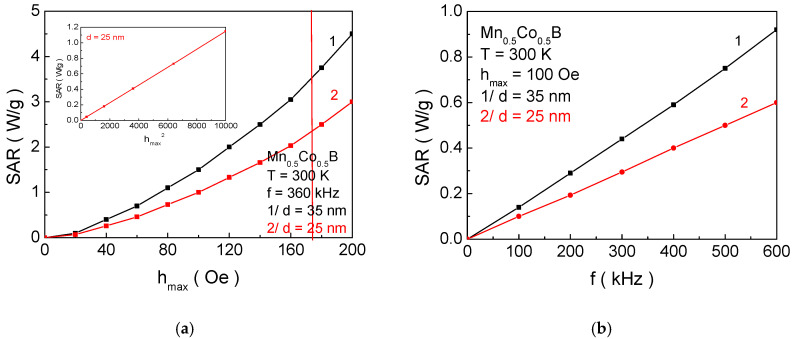
Dependence of the SAR coefficient on (**a**) the amplitude of *h_max_* of the external electromagnetic field at *f* = 360 and (**b**) the frequency of the external electromagnetic field at *h_max_ =* 100 Oe for Mn_0.5_Co_0.5_B at *T* = 300 K. The red vertical curve defines the biologically safe value of *h_max_.f* < 6.2 × 10^7^ Oe/s. In the inset the SAR is plotted as a function of $h_{nax}^{2}$.

Figure 8 shows the dependence of the SAR values on the amplitude *h_max_* and frequency *f* of the external alternating electromagnetic field for a given nanoparticle size *d* and a specific dopant concentration *x*, suitable for SCMH. A quadratic dependence of the thermal efficiency on *h_max_* (see inset in Figure 8a) and a linear dependence on *f* have been established. This means that it is more effective to vary the amplitude of the field at lower frequency values, as this leads to higher SAR values. The obtained results are in good agreement with experimental data [19,59] and demonstrate the adequacy of the microscopic model we used, the approximations made, and the accuracy of the calculations.

**Figure 9:**
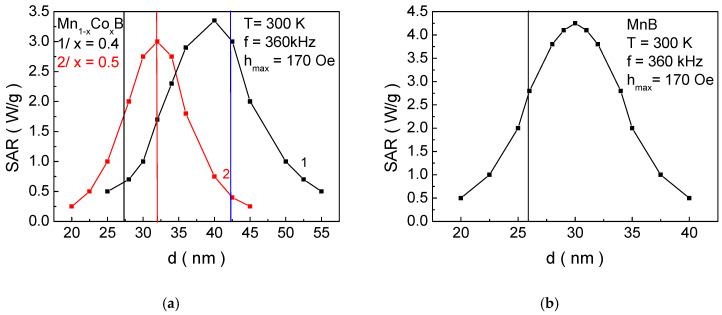
Dependence of SAR on the size of NPs: (**a**) curve 1 Mn_0.6_Co_0.4_B; curve 2 Mn_0.5_Co_0.5_B; (**b**) MnB at *h_max_* = 170 Oe and *f* = 360 kHz for *T* = 300 K. The vertical lines represent the sizes of NPs that are biocompatible and meet the requirements for SCMH.

Figure 9a shows the dependence of the SAR coefficient on the NP size *d* with the structural formulas: curve 1—Mn_0.6_Co_0.4_B; curve 2—Mn_0.5_Co_0.5_B; and Figure 9b/for MnB. The vertical lines represent the NP sizes that have the magnetic phase transition temperature within the biocompatible range. The intersection points (with the same color as the curves) provide the value of the SAR, i.e., their thermal efficiency. It is evident that these particles do not exhibit maximum thermal efficiency. For Mn_0.6_Co_0.4_B, the maximum SAR value is obtained for a NP size of 40.3 nm (SAR_max_ = 3.46 W/g), for Mn_0.5_Co_0.5_B—32.2 nm (SAR_max_ = 3.05 W/g) (this is the NP size that is biocompatible for MH), and for MnB—29.9 nm (SAR_max_ = 4.21 W/g). This means that each particle satisfying the conditions for MH will not have the maximum efficiency of heat transfer to tissues. On the other hand, the particles Mn_0.6_Co_0.4_B and MnB, for which the SAR is maximum, have magnetic phase transition temperatures of 427 K and 364 K, respectively, making them unacceptable for SCMH (despite the higher SAR values compared to Mn_0.5_Co_0.5_B—32.2 nm (SAR_max_ = 3.05 W/g)).

Thus, fulfilling the necessary condition for the biocompatibility of the magnetic phase transition temperature, we obtain the following results: Mn_0.6_Co_0.4_B—SAR = 0.65 W/g; Mn_0.5_Co_0.5_B—SAR = SAR_max_ = 3.05 W/g; and MnB—SAR = 3.82 W/g. In [9], for particles with a size of 25 nm, the experimentally determined SAR value for FeB is 1.8–2.0 W/g, which are in alignment with our theoretical results.

In Figure 9a, with the increase in the concentration of dopant Co ions *x* for Mn_1−x_Co_x_B, the peak of the SAR curve shifts towards smaller values *d_max_* of the NP size. It can be observed that, for a fixed size, when *d* < *d_max_*, decreasing *x* leads to a reduction in the SAR value (see the vertical black line), while for *d* > *d_max_*, decreasing *x* leads to an increase in the SAR (see the vertical blue line). This can be qualitatively explained as follows: The reduction in x leads to a decrease in the magnetic crystalline anisotropy and a reduction in the potential barrier between the two directions of magnetization. For small particles *d* < *d_max_*, the energy of magnetic anisotropy and thermal energy becomes comparable, and the spins easily transition between the two energetically stable states. This results in a significant reduction in the relaxation time, and the area of the dynamic hysteresis curves decreases;, i.e., the heat transfer reduces. For larger particles *d* > *d_max_*, the increase in magnetic crystalline anisotropy (i.e., with the increase in dopant Co ions) causes the spins to be practically blocked in one of the two equilibrium states, performing weak precession around it. This leads to a decrease in the area of the hysteresis curves and consequently to a reduction in the SAR.

Based on the performed numerical calculations, the following parameters for NPs with the structural formula Mn_1−x_X_x_B (X = Co, Fe), suitable for SCMHT, have been determined (see Table 2).

### 3.3. Bulk (Mn_1−x_X_x_)_2_ B (X = Co, Fe)

**Figure 10:**
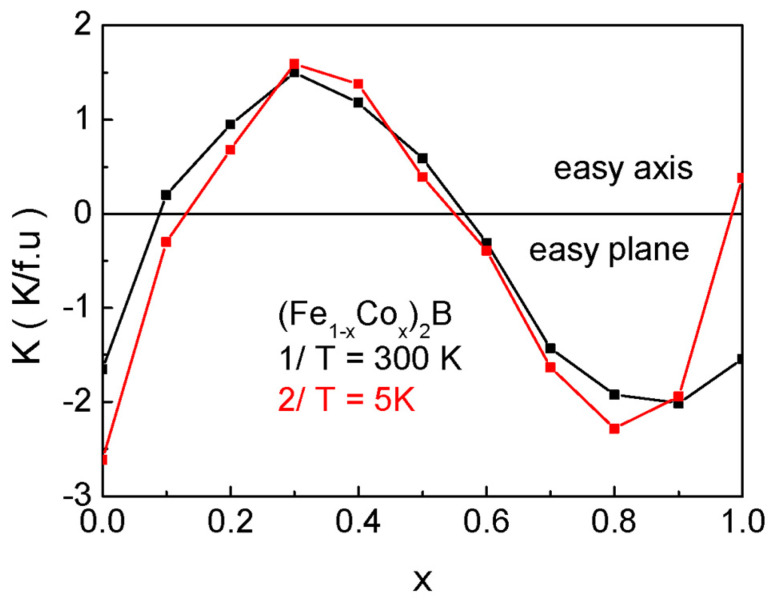
Dependence of the magnetocrystalline anisotropy on the concentration of doped Co ions for bulk (Fe_1−x_Co_x_)_2_B at 1/*T* = 300 K and 2/*T* = 5 K. The data for constructing the dependence are from [41,48].

For the numerical calculations for bulk (Fe_1−x_Co_x_)_2_B, we will use the following model parameters (see Table 3):

The exchange interaction constants in undoped compounds are calculated within the mean field method using the following expression: ${\;J}^{X-X}=\frac{3\;{T_{C}}^{X}}{2zS\left(S+1\right)}\;$(X = Fe, Co) in units of K, where *T_C_* is the Curie temperature, as given in column 4 of Table 3, and *z* is the number of nearest neighbors. For doped samples, the exchange interaction between Fe ions and doped Co ions is as follows: *J*^Fe-Co^ = 57.45 K. As highlighted in Section 2, there is a strong dependence of the value and sign of the magnetocrystalline anisotropy on the doping level with Co ions. Therefore, in the numerical calculations, we will use the data for *K* from Figure 10.

In presenting the results for (Fe_1−x_Co_x_)_2_B, we will follow the same sequence as for Mn_1−x_X_x_B.

**Figure 11:**
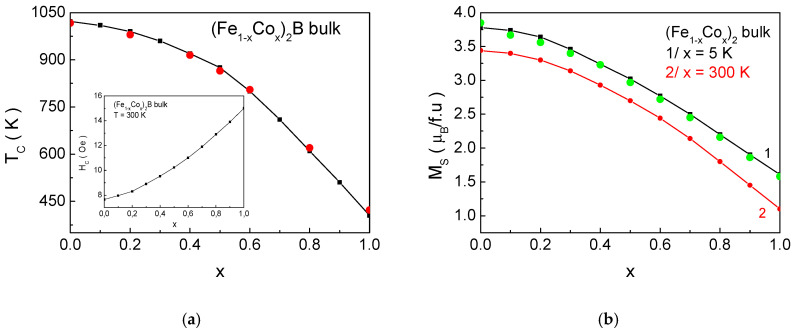
Dependency of (**a**) *T_C_* and (**b**) *Ms* as follows: curve 1 for *T* = 5 K and curve 2 for *T* = 300 K as a function of the doping concentration *x* for (Fe_1−x_Co_x_)_2_B bulk samples. The insets show the dependence of coercivity on the doping level *x* for *T* = 300 K. The red points in Figure 11a/represent experimental values for *T_C_* obtained from the studies of [41,60]. The green points in Figure 11b/represent experimental values for *M_s_* from [41,61].

Figure 11 presents the dependence of the magnetic phase transition temperature *T_C_*, the saturation magnetization *M_s_*, and the coercivity *H_c_* as a function of the Co ion concentration *x* for (Fe_1−x_Co_x_)_2_B bulk samples. The obtained results show good quantitative agreement with experimental data published in the studies of [41,60,61]. As discussed in Section 3.2, there is a noticeable sensitivity to the degree of doping. This provides the opportunity to manipulate *T_c_*, *H_c_*, and *M_s_* over a wide range by adjusting the composite composition of the alloys, in accordance with potential technological applications.

### 3.4. (Mn_1−x_X_x_)_2_ B (X = Co, Fe) Nanoparticles

**Figure 12:**
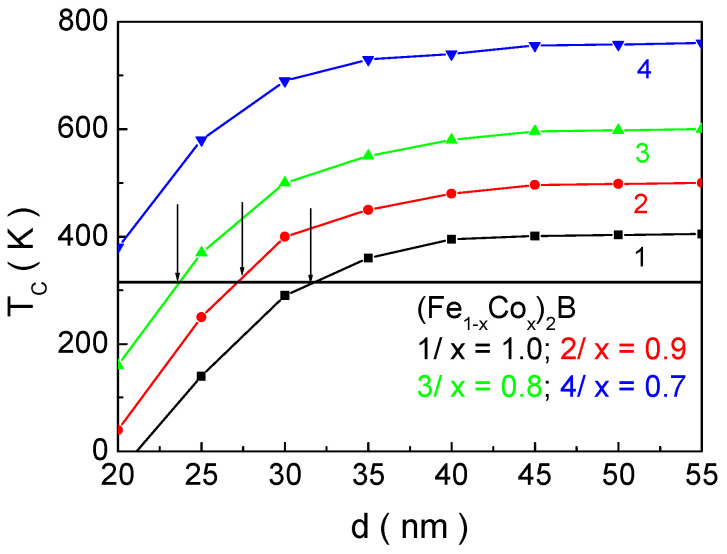
Dependence of the magnetic phase transition temperature *T_C_* on the size *d* of the nanoparticles for (Fe_1−x_Co_x_)_2_B at different concentrations of the dopant ion *x*.

Figure 12 shows the possibility of using an “additional degree of freedom” to change the temperature of the magnetic phase transition within a wide range, namely, the so-called size and surface effects. These effects are manifested in a drastic change in the magnetic properties of NPs by altering the surface-to-volume ratio. This ratio rises with a decrease in the NP size and leads to an increased influence of the surface on the properties of the system (this influence is discussed in detail in Section 3.2). As the NP size *d* decreases, *T_C_* decreases, too, and, for certain concentration values of Co ions, the necessary condition for the applicability of these particles for SCMH is fulfilled. The horizontal black line represents the boundary for *T_C_* (315 K), which must not be exceeded. The intersection points of the *T_C_*(*d*) curves at different concentrations *x* of the doping element allow the determination of the structural formula and particle size that satisfy the necessary conditions for the phase transition temperature and the dimensions suitable for in vitro and in vivo applications of MNPs in SCMH. Based on this approach, we have identified that NPs with the following parameters are suitable candidates for SCMH: (Fe_0.2_Co_0.8_)_2_B—*d* = 22.0 nm; (Fe_0.1_Co_0.9_)_2_B—*d* = 26.3 nm; and Co_2_B—*d* = 31.8 nm.

**Figure 13:**
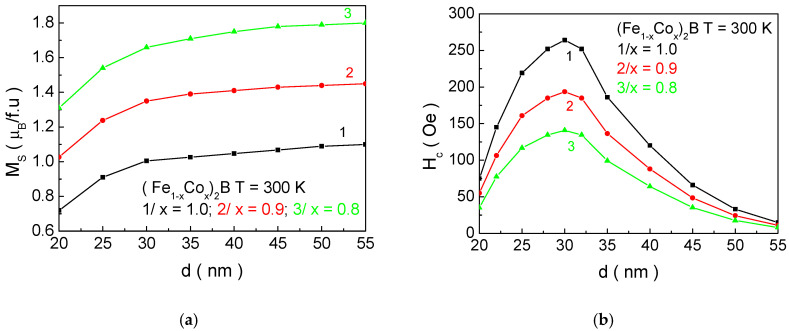
Dependence of (**a**) the saturation magnetization *M_s_* and (**b**) the coercivity *H_c_* as a function of the particle size *d* for (Fe_1−x_Co_x_)_2_B at different concentrations *x* of the doping Co ions: 1/*x* = 1.0; 2/*x* = 0.9 and 3/*x* = 0.8.

Figure 13 presents the dependence of the saturation magnetization *M_s_* and the coercivity *H_c_* as a function of the particle size *d* for NPs with Co concentrations suitable for MH. These numerical calculations allow us to determine the *M_s_* and *H_c_* values for NPs with biologically acceptable Curie temperatures *T_C_*. It was found that *M_s_* and *H_c_* have the following values for (Fe_0.2_Co_0.8_)_2_B (*d* = 22.0 nm)—1.52 μB/f.u; 75 Oe; (Fe_0.1_Co_0.9_)_2_B (*d* = 26.3 nm)—1.26 μB/f.u; 173 Oe; and Co_2_B (*d* = 31.8 nm)—0.98 μB/f.u; 268 Oe. A comparison of the *M_s_* and *H_c_* values for X_2_B and XB (X = Mn, Fe, Co) compounds shows that the saturation magnetization *M_s_* for X_2_B is higher compared to XB, while the coercivity *H_c_* is lower for X_2_B than for XB. In other words, the X_2_B compounds are magnetically “softer” than XB, making the former more suitable for MH applications (see Introduction). This conclusion was experimentally confirmed in the study of [17].

**Figure 14:**
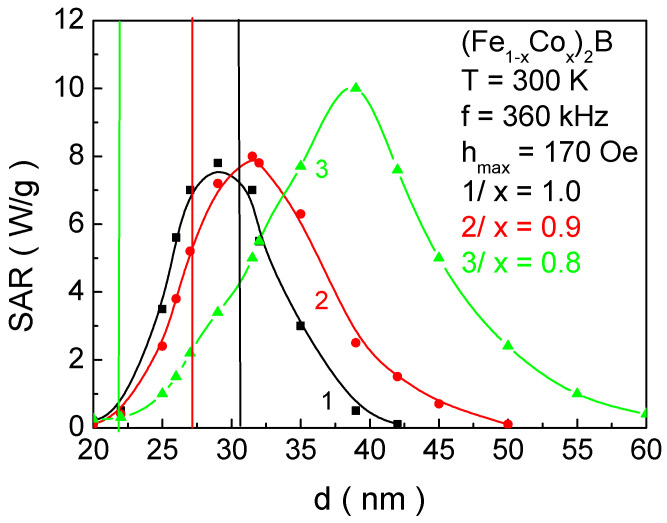
Dependence of the SAR on the NP size *d* for 1/Co_2_B; 2/(Fe_0.1_Co_0.9_)_2_B; 3/(Fe_0.2_Co_0.8_)_2_B at *h*_max_ = 170 Oe and *f* = 360 kHz at *T* = 300 K. The vertical lines indicate the NP sizes that are biocompatible and meet the requirements for SCMH.

Figure 14 presents the dependence of the SAR coefficient on the NP size *d* with the following structural formulas: curve 1—Co_2_B; curve 2—(Fe_0.1_Co_0.9_)_2_B; and curve 3(Fe_0.2_Co_0.8_)_2_B. The vertical lines indicate the NP sizes whose Curie temperatures fall within the biocompatible range. The intersection points (marked in the same color as the curves) determine the corresponding SAR values, i.e., their thermal efficiency. It is evident that these particles do not achieve maximum thermal efficiency. For Co_2_B, the maximum SAR value is reached at an NP size of 28.9 nm (SAR_max_ = 7.12 W/g); for (Fe_0.1_Co_0.9_)_2_B, at 32.4 nm (SAR_max_ = 8.01 W/g) (this size is also biocompatible for MH); and for (Fe_0.2_Co_0.8_)_2_B at 39.7 nm (SAR_max_ = 9.93 W/g) (in this case, the particle size exceeds the limits suitable for in vitro and in vivo applications).

On the other hand, NPs with the Co_2_B structural formula and the size *d* corresponding to the SAR maximum have Curie temperatures of 298 K and 410 K, respectively. This makes them unsuitable for MH.

Thus, by fulfilling the necessary condition for the biocompatibility of the magnetic phase transition temperature, we obtain the following results: (Fe_0.2_Co_0.8_)_2_B—SAR = 0.25 W/g; (Fe_0.1_Co_0.9_)_2_B—SAR = 5.2 W/g; and Co_2_B—SAR = 7.1 W/g.

Based on the numerical calculations performed, the following parameters have been determined for NPs with the structural formula (Fe_1−x_Co_x_)_2_B, suitable for MH applications (see Table 4).

## 4. Conclusions

Based on a microscopic model and employing Green’s function technique, we investigate NPs with structural formulas Mn_1−x_X_x_B (X = Fe, Co) and (Fe_1−x_Co_x_)_2_B, with sizes up to 35 nm and phase transition temperatures *T_C_* ≈ 315 K. These parameters make the NPs suitable for in vivo and in vitro medical applications in cancer treatment—specifically SCMH. The heterogeneous structure of the MNPs is taken into account by considering the differences in exchange magnetic interactions and magnetocrystalline anisotropy between the core and the surface. Numerical results for the dependence of the Curie temperature *T_C_*, saturation magnetization *M_s_*, and coercivity *H_C_* on NP size and dopant ion concentration *x* are explained on a microscopic level. A set of particles that meets the biocompatibility requirements—preventing the overheating of healthy tissues and minimizing patient exposure to high-frequency electromagnetic fields—is identified. The characteristics of these NPs are summarized in Table 2 and Table 4. For these particles, the SAR, a measure of heating efficiency, is calculated. The adequacy of the applied microscopic model is demonstrated by both quantitative and qualitative agreement with numerous experimental results.

## Figures and Tables

**Table 1:** Main model parameters for the numerical calculations of bulk Mn_1−x_X_x_B (X = Co, Fe).

Compound	S (X^3+^)	*J*^X-X^ (K)	*K*^X^ (K/f.u)	${Tc}^{X}$ (K)
MnB	2	35.13	3.19 [31]	546 [54]
FeB	5/2	25.28	0.93 [31]	590 [18]
CoB	2	29.34	10.11 [31]	477 [55]

**Table 2:** Parameters of NPs with the structural formula Mn_1−x_X_x_B (X = Co, Fe), suitable for SCMHT for *f =* 360 kHz.

Compound	d (nm)	M_S_ (µ_B_/f.u)	H_C_ (Oe)	SAR (W/g)
Mn_0.6_Co_0.4_B	27.1	0.32	278	0.65
Mn_0.5_Co_0.5_B	32.2	0.23	352	3.05
MnB	26.3	0.95	210	2.82

**Table 3:** Model parameters for numerical calculations of bulk (Fe_1−x_Co_x_)_2_B.

Compound	S (X^3+^)	J^X-X^ (K)	Tc (K)
Fe_2_B	2	64.19	1027 [60]
Co_2_B	3/2	42.20	422 [60]

**Table 4:** Parameters of NPs with the structural formula (Fe_1−x_Co_x_)_2_B, suitable for SCMH for *f =* 360 kHz.

Compound	d (nm)	M_S_ (µ_B_/f.u)	H_C_ (Oe)	SAR (W/g)
(Fe_0.2_Co_0.8_)_2_B	22.0	1.52	75	0.25
(Fe_0.1_Co_0.9_)_2_B	26.3	1.26	173	5.2
Co_2_B	31.8	0.98	269	7.1

## Data Availability

The original contributions presented in this study are included in the article. Further inquiries can be directed to the corresponding author.

## Appendix A

The calculated Green’s functions for ${Mn}_{1-x}X_{x}B$, where X = Fe, Co, in the random phase approximation are as follows:

(A1) $${G_{ij,E}}^{Mn-Mn}=\frac{\left(\frac{i}{\pi }\right)[E-{{\tilde{E}}_{ij}}^{X-X}]\langle S_{i}^{Mn,z}\rangle \delta_{ij}}{\left\{\left[E-{{\tilde{E}}_{ij}}^{X-X}\right]\left[E-{{\tilde{E}}_{ij}}^{Mn-Mn}\right]-{{\tilde{E}}_{ij}}^{X-Mn}{{\tilde{E}}_{ij}}^{Mn-X}\right\}};\;{G_{ij,E}}^{X-X}=\frac{\left(\frac{i}{\pi }\right)[E-{{\tilde{E}}_{ij}}^{Mn-Mn}]\langle S_{i}^{X,z}\rangle \delta_{ij}}{\left\{\left[E-{{\tilde{E}}_{ij}}^{X-X}\right]\left[E-{{\tilde{E}}_{ij}}^{Mn-Mn}\right]-{{\tilde{E}}_{ij}}^{X-Mn}{{\tilde{E}}_{ij}}^{Mn-X}\right\}};\;{G_{ij,E}}^{X-Mn}=\frac{\left(\frac{i}{\pi }\right){{\tilde{E}}_{ij}}^{X-Mn}\langle S_{i}^{Mn,z}\rangle \delta_{ij}}{\left\{\left[E-{{\tilde{E}}_{ij}}^{X-X}\right]\left[E-{{\tilde{E}}_{ij}}^{Mn-Mn}\right]-{{\tilde{E}}_{ij}}^{X-Mn}{{\tilde{E}}_{ij}}^{Mn-X}\right\}};\;{G_{ij,E}}^{Mn-X}=\frac{\left(\frac{i}{\pi }\right){{\tilde{E}}_{ij}}^{Mn-X}\langle S_{i}^{X,z}\rangle \delta_{ij}}{\left\{\left[E-{{\tilde{E}}_{ij}}^{X-X}\right]\left[E-{{\tilde{E}}_{ij}}^{Mn-Mn}\right]-{{\tilde{E}}_{ij}}^{X-Mn}{{\tilde{E}}_{ij}}^{Mn-X}\right\}};$$

where ${{\tilde{E}}_{ij}}^{\alpha -\beta }={E_{ij}}^{\alpha -\beta }-i{\gamma_{ij}}^{\alpha -\beta }$ with α,β=Mn,X, ${E_{ij}}^{\alpha -\beta }$ is the interaction energy between the i-th and j-th spins, and ${\gamma_{ij}}^{\alpha -\beta }$ is the damping.

For the interaction energy ${E_{ij}}^{\alpha -\beta }$ between the *i*-th and *j*-th spins, we obtain the following:

$${E_{ij}}^{Mn-Mn}=\frac{2}{N}\sum_{k}J_{ikeff}^{Mn-Mn}\langle S_{k}^{Mn,z}\rangle \delta_{ij}-2J_{ijeff}^{Mn-Mn}\langle S_{i}^{Mn,z}\rangle \delta_{ij}-\;\frac{2}{N}\sum_{k}J_{ik}^{Mn-Mn}\langle S_{k}^{Mn,z}\rangle \delta_{ij}--g\mu_{B}h\delta_{ij};$$

$${E_{ij}}^{X-X}=\frac{2}{N}\sum_{k}J_{ikeff}^{X-X}\langle S_{k}^{X,z}\rangle \delta_{ij}-2J_{ijeff}^{X-X}\langle S_{i}^{X,z}\rangle \delta_{ij}-\frac{2}{N}\sum_{k}J_{ik}^{X-X}\langle S_{k}^{X,z}\rangle \delta_{ij}-g\mu_{B}h\delta_{ij};$$

$${E_{ij}}^{X-Mn}=2J_{ij}^{X-Mn}\langle S_{i}^{X,z}\rangle \delta_{ij};$$

$${E_{ij}}^{Mn-X}=2J_{ij}^{Mn-X}\langle S_{i}^{Mn,z}\rangle \delta_{ij};$$

where $J_{ijeff}^{Mn-Mn}=J_{ij}^{Mn-Mn}+K_{i}^{Mn}\delta_{ij}$; $J_{ijeff}^{X-X}=J_{ij}^{X-X}+K_{i}^{X}\delta_{ij}$.

Only those expressions for the damping ${\gamma_{ij}}^{\alpha -\beta }$ are provided for which the law of energy conservation is satisfied.

(A2) $$\gamma_{ij}^{Mn-Mn}=\frac{4\pi }{N}\sum_{l}{\left(J_{il}^{Mn-Mn}\right)}^{2}{E_{ij}}^{X-Mn}\{n_{l}^{Mn-Mn}*\left[2\langle S_{k}^{Mn,z}\rangle +n_{l}^{Mn-Mn}+n_{i}^{Mn-Mn}\right]-\;n_{j}^{Mn-Mn}*n_{l}^{Mn-Mn}\}\delta \left(E_{l}^{Mn-Mn}+E_{j}^{Mn-Mn}-E_{i}^{Mn-Mn}-E_{l}^{Mn-Mn}\right);$$

(A3) $$\gamma_{ij}^{X-X}=\frac{4\pi }{N}\sum_{l}{\left(J_{il}^{X-X}\right)}^{2}\langle S_{k}^{X}\rangle \left\{n_{l}^{X-X}*\left[2\langle \langle S_{k}^{X,z}\rangle +n_{l}^{X-X}+n_{i}^{X-X}\right]-n_{j}^{X-X}*n_{l}^{X-X}\right\}*\;\delta \left(E_{l}^{Mn-Mn}+E_{j}^{Mn-Mn}-E_{i}^{Mn-Mn}-E_{l}^{Mn-Mn}\right)\;;$$

(A4) $$\gamma_{ij}^{Mn-X}=\frac{4\pi }{N}\sum_{l}{\left(J_{il}^{Mn-X}\right)}^{2}n_{i}^{Mn-X}*n_{l}^{Mn-X}\;\delta \left(E_{i}^{Mn-X}-E_{l}^{Mn-X}\right)\delta_{lj};$$

(A5) $$\gamma_{ij}^{X-Mn}=\frac{4\pi }{N}\sum_{l}{\left(J_{il}^{X-Mn}\right)}^{2}n_{i}^{X-Mn}*n_{l}^{X-Mn}\;\delta \left(E_{i}^{X-Mn}-E_{l}^{X-Mn}\right)\delta_{lj};$$

where $n_{i}^{\alpha -\beta }=\langle S_{i}^{\alpha ,-}S_{j}^{b,+}\rangle$ are the spin correlation functions for α, β = Mn, X, which are calculated using the spectral theorem [50].

$$\langle S_{i}^{Mn,-}S_{j}^{Mn,+}\rangle =\frac{1}{2\pi }\left[\Omega^{Mn-Mn}\left(E_{ij}^{1}\right)+\Omega^{Mn-Mn}\left(E_{ij}^{2}\right)\right]\langle S_{i}^{Mn,z}\rangle \delta_{ij}$$

$$\langle S_{i}^{X,-}S_{j}^{X,+}\rangle =\frac{1}{2\pi }\left[\Omega^{X-X}\left(E_{ij}^{1}\right)+\Omega^{X-X}\left(E_{ij}^{2}\right)\right]\langle S_{i}^{X,z}\rangle \delta_{ij}$$

$$\langle S_{i}^{Mn,-}S_{j}^{X,+}\rangle =-\langle S_{i}^{X,-}S_{j}^{Mn,+}\rangle =\frac{J_{ij}^{Mn-X}}{\pi }\left[\Lambda \left(E_{ij}^{1}\right)+\Lambda \left(E_{ij}^{2}\right)\right]\langle S_{i}^{Mn,z}\rangle \langle S_{j}^{X,z}\rangle$$

where

$$\Omega^{Mn-Mn}\left(E\right)=\frac{\left(2E-\Delta_{ij})(E-{E_{ij}}^{Mn-Mn}\right)}{exp(\frac{E}{k_{B}T})-1};$$

$$\Omega^{X-X}\left(E\right)=\frac{\left(2E-\Delta_{ij})(E-{E_{ij}}^{X-X}\right)}{exp(\frac{E}{k_{B}T})-1};$$

$$\Lambda \left(E\right)=2E-\Delta_{ij};\;\Delta_{ij}=({E_{ij}}^{Mn-Mn}+{E_{ij}}^{X-X})$$

as $E_{ij}^{1,2}$ is determined from the poles of Green’s functions (A1):

$$E_{ij}^{1,2}=0.5({E_{ij}}^{Mn-Mn}+{E_{ij}}^{X-X})\pm {\left[{\left({E_{ij}}^{Mn-Mn}-{E_{ij}}^{X-X}\right)}^{2}-\;4{E_{ij}}^{X-Mn}{E_{ij}}^{Mn-X}\right]}^{1/2}.$$

## Appendix B

The calculated expressions for $\omega_{ij}(S^{X}$) beyond the random phase approximation (RPA) are as follows:

$$\omega_{ij}\left(S^{Mn}\right)=\frac{1}{\langle S_{i}^{Mn,z}\rangle }\{\frac{2}{N}\sum_{k}J_{ikeff}^{Mn-Mn}\left[\langle S_{k}^{Mn,+}S_{j}^{Mn,-}\rangle -2\langle S_{k}^{Mn,z}S_{j}^{Mn,z}\rangle \right]-2J_{ijeff}^{Mn-Mn}\;*\;\left[\langle S_{k}^{Mn+}S_{j}^{Mn,-}\rangle -\langle S_{k}^{Mn,z}S_{j}^{Mn,z}\rangle \right]-\frac{2}{N}\sum_{k}J_{ik}^{Mn-X}\left[\langle S_{k}^{X,+}S_{j}^{Mn,-}\rangle -2\langle S_{k}^{X,z}S_{j}^{Mn,z}\rangle \right]-\;2J_{ij}^{Mn-X}\left[\langle S_{i}^{X,+}S_{j}^{Mn,-}\rangle -\langle S_{i}^{X,z}S_{j}^{Mn,z}\rangle \right]-g\mu_{B}h\langle S_{j}^{Mn,z}\rangle \delta_{ij}\};$$

$$\omega_{ij}\left(S^{X}\right)=\frac{1}{\langle S_{i}^{Mn,z}\rangle }\{\frac{2}{N}\sum_{k}J_{ikeff}^{X-X}\left[\langle S_{k}^{X,+}S_{j}^{X,-}\rangle -2\langle S_{k}^{X,z}S_{j}^{X,z}\rangle \right]-2J_{ijeff}^{X-X}\;*\left[\langle S_{k}^{X+}S_{j}^{X,-}\rangle -\;\langle S_{k}^{X,z}S_{j}^{X,z}\rangle \right]-\frac{2}{N}\sum_{k}J_{ik}^{X-Mn}\left[\langle S_{k}^{X,+}S_{j}^{Mn,-}\rangle -2\langle S_{k}^{Mn,z}S_{j}^{X,z}\rangle \right]-2J_{ij}^{X-Mn}\left[\langle S_{i}^{Mn,+}S_{j}^{X,-}\rangle -\;\langle S_{i}^{Mn,z}S_{j}^{X,z}\rangle \right]-g\mu_{B}h\langle S_{j}^{X,z}\rangle \delta_{ij}\}.$$

## Appendix C

From the expression (3) for the average absorbed power P of a magnetic nanoparticle, we obtain the following:

(A6) $$\begin{array}{cc} P=4\sum_{ij}\frac{{2g}^{2}\mu_{B}^{2}}{\pi } & \;*\frac{EA_{2}}{E^{2}+A_{1}}*\gamma_{tot}\\ & \;*[\frac{\left\{\left[E-{E_{ij}}^{X-X}+2{E_{ij}}^{X-Mn}\right]\langle S_{i}^{Mn,z}\rangle \right\}}{{\left[\Lambda \left(E\right)\right]}^{2}+4E^{2}{\left(\gamma_{tot}\right)}^{2}}\\ & \;+\frac{\left\{\left[E-{E_{ij}}^{Mn-Mn}+2{E_{ij}}^{Mn-X}\right]\langle S_{i}^{X,z}\rangle \rangle \right\}}{{\left[\Lambda \left(E\right)\right]}^{2}+4E^{2}{\left(\gamma_{tot}\right)}^{2}}]E^{2}{\left(h_{max}\right)}^{2} \end{array}$$

where $\Lambda \left(E\right)=\frac{{\left(E^{2}-A_{1}\right)}^{2}-E^{2}A_{2}^{2}-E^{2}{\left(\gamma^{\prime }\right)}^{2}+A_{3}^{2}}{E^{2}+A_{1}}$, $\gamma_{tot}=\gamma^{\prime }+\frac{A_{2}A_{3}}{E^{2}+A_{1}}$.

The expressions for $A_{1},\;A_{2},\;A_{3},$ and γ′ have the following form:

$$A_{1}={E_{ij}}^{X-X}{E_{ij}}^{Mn-Mn}-{E_{ij}}^{X-Mn}{E_{ij}}^{Mn-X}-\gamma_{ij}^{Mn-Mn}\gamma_{ij}^{X-X}-\gamma_{ij}^{Mn-X}\gamma_{ij}^{X-Mn};$$

$$A_{2}={E_{ij}}^{Mn-Mn}+{E_{ij}}^{X-X};$$

$$A_{3}={E_{ij}}^{X-X}\gamma_{ij}^{Mn-Mn}+{E_{ij}}^{Mn-Mn}\gamma_{ij}^{X-X}+{E_{ij}}^{X-Mn}\gamma_{ij}^{Mn-X}+{E_{ij}}^{Mn-X}\gamma_{ij}^{X-Mn};$$

$$\gamma^{\prime }=\gamma_{ij}^{Mn-Mn}+\gamma_{ij}^{X-X}.$$

$\gamma_{tot}$ determines the damping in the system.

In expression (A6), the thermal efficiency is expressed solely based on the microscopic characteristics of the spin elementary excitations and their damping, i.e., through magnetic exchange interactions and magnetic crystalline anisotropy.
